# Correlation Factor Study of Small Punch Creep Test and Its Life Prediction

**DOI:** 10.3390/ma9100796

**Published:** 2016-09-24

**Authors:** Cheng Wen, Tong Xu, Kaishu Guan

**Affiliations:** 1 School of Mechanical and Power Engineering, East China University of Science and Technology, No. 130 Meilong Street, Xuhui District, Shanghai 200030, China; wen904437954@163.com; 2 China Special Equipment Inspection & Research Institute, Building 2, Hepingjie, Xiyuan, Chaoyang District, Beijing 100029, China; xutong@csei.org.cn

**Keywords:** small punch creep test, uniaxial creep test, correlation factor, life prediction

## Abstract

A small punch test is one of the innovative methods that can be used to evaluate the properties of a material without destructive harm to the in-service component. Conventionally identifying material properties by a uniaxial test is widely applied to engineering. How the properties obtained from a small punch test can be applied with the same utility has been a goal. In 2006, European Code of Practice (CoP) of small punch tests was first released, in which the correlation factor, k_sp_, was introduced to bridge the gap between the above methods. The author investigates the relationship between a uniaxial creep text and a small punch creep test by exploring the correlation factor k_sp_. Various sets of experiments and a comparative study of the conventional uniaxial creep test and small punch creep test were carried out. Methods including Norton, Larson-Miller and Time versus Stress relation were employed to identify the value of k_sp_. Different k_sp_ values were found in different materials, which indicate that k_sp_ values of materials need to be identified separately. In addition, the life prediction of a small punch creep test was carried out and the results of the life prediction predict a reasonable accuracy, which indicates that the small punch creep test is a reliable method for life prediction.

## 1. Introduction

The development of the modern industrial history is characterized by the continually increasing operating temperature of the industrial equipment and machineries. The operating temperature changes from about 160 °C in the boiler of the steam engine to 1800 °C in the aircraft engine turbine [1]. Modern engineering materials are confronted with increasingly strict operating conditions, where complex geometry structure, high operating pressure, high stress state and elevated temperature are challenging the reliability of the material. During the service period, the degradation of material, which includes a change of the microstructure, the gathering of precipitation and carbide spheroidization and coalescence, could result in the premature occurrence of failure. Therefore, the determination of the properties of material at high temperature and the evaluation of residual life are crucial. In addition, these two fields are frequently studied by researchers from all over the world.

Typically, the mechanical behavior of a specific material can be obtained from the conventional uniaxial test. However, in order to carry out the uniaxial test, a large amount of material is needed to make the uniaxial specimen. In situations where there is a need for the evaluation of an in-service component, conventional tests are unable to fulfil this task due to massive destruction to the equipment. The need of a mini-invasive assessing approach advances the development of small sample test techniques. Among all the small sample test techniques, the small punch test is considered as the most promising one since first introduced in the early 1980s by Manaham et al. at MIT [2], as an effective method to assess the degradation of mechanical properties of materials after nuclear radiation [3,4,5,6]. To conduct a small punch test, only a small size of material is needed to make the specimen (8–10 mm × 10 mm × 0.5 mm). Such a small size enables evaluating properties of materials un-destructively. J. D. Parker and J. D. James et al. [7] pointed out that the small punch test could be applied to obtain the creep properties of materials at an elevated temperature. 

However, it is the conventional uniaxial creep test results, i.e., Norton parameters, which are applied to the engineering design. For the aim of applying the results obtained from the small punch creep test, a correlation needs to be established between the small punch creep test and the uniaxial creep test. In the uniaxial test, the test specimen is only subjected to single stress state, which is tensile stress. While in the small punch creep test, the test specimen bends while the punch ball moves downwards. Thus, in this situation, the punch imposes a three state stress to the test specimen. The discrepancy in stress state leads to the gap between the results of the above two methods.

In order to interpret the small punch creep test curve, a variety of theoretical models were proposed by researchers. Among all these theoretical models, the membrane stretch model devised by Chakrabarty [8] was most widely used.

**Figure 1:**
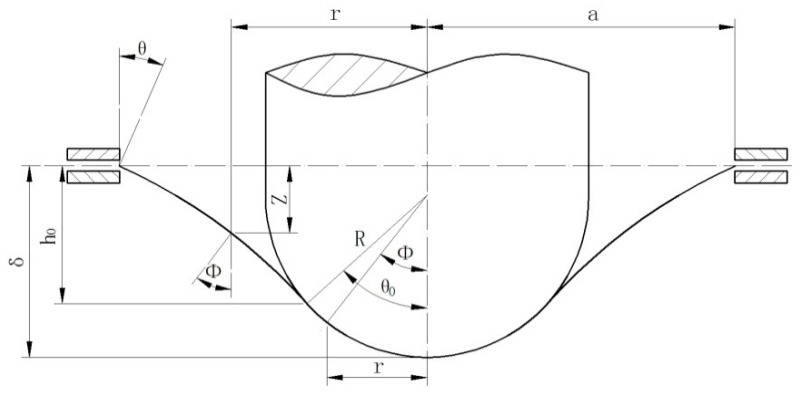
Geometry of the Chakrabarty membrane stretch model.

The material model of Chakrabarty is shown in Figure 1. It was assumed that the specimen is a deformable circular blank and that the punch is regarded as a hemispherical rigid body. Overall, the small punch specimen deforms under the membrane stress alone.

### 1.1. Strain of the Small Punch Test

In this model, the relation between the deflection of the lower face of the specimen and the strain was studied. The general equation of compressive thickness strain is:

(1) $$\varepsilon =2\ln\frac{2\left(1+\cos\emptyset \right)\left(1+\cos\theta \right)}{{\left(1+{\cos\theta }_{0}\right)}^{2}}$$

Maximum strain at the centre of the specimen is:

(2) $$\varepsilon =2\ln\frac{2\left(1+\cos\theta \right)}{{\left(1+{\cos\theta }_{0}\right)}^{2}}$$

It was proved by Chakrabarty that the circumferential strain $\varepsilon_{c}$ equals the radial strain $\varepsilon_{r}$. As the volume of the specimen is assumed as incompressible, the compressive thickness strain turns out to be the equivalent strain

Yang and Wang [9] studied the Chakrabarty membrane stretch model and gave out the relation between the equivalent strain and central deflection. By setting $\theta_{0}$ to the values in the range from 0° to 90°, together with fitting the values of strain and deflection by a ploy–nominal expression, the equation between strain and central deflection is:

(3) $$\varepsilon =0.2111\delta^{2}+0.329\delta$$

### 1.2. Equivalent Stress of the Small Punch Creep Test

Current studies in the field of the small punch creep test focus on finding out the relationship between the small punch creep test and the uniaxial creep test. With the derived strain from the Chakrabarty model, the other important factor needed is the stress of the small punch creep test. It needs to be noted that the mentioned stress (σ) is not the specific small punch creep test stress. The stress of the small punch specimen varies in different locations; the equivalent stress (σ) is the equivalent uniaxial creep stress. Since the strain and equivalent stress of the small punch test lies in the core of the correlation, a relationship between the applied load F_sp_ of the small punch test and the equivalent stress (σ) of the uniaxial creep test must be established. 

Derived from an equilibrium of the Chakrabarty model and through mathematic deduction, the ratio between load $F_{\mathrm{sp}}$ and equivalent stress σ is given as:

(4) $$\frac{F_{\mathrm{sp}}}{\sigma }=2\pi \;\times \;{\mathrm{Rsin}}^{2}\theta_{0}\;\times \;t$$

where the t stands for the thickness of the specimen.

Yang and Wang [9] studied the ratio between load F_sp_ and equivalent stress σ; the following equation was derived:

(5) $$\frac{F_{\mathrm{sp}}}{\sigma }=0.6521\delta^{-1.1442}$$

However, the above Equations (4) and (5) are in inexplicit form; they are either the function of the angle $\theta_{0}$ or the deflection δ. Firstly, in 2006, bringing together the previous studies, an explicit form of the equation was introduced by the European standard “Europe Code of Practice: A Code of Practice for Small Punch Creep Testing” [10]. The equation is expressed as a function of the geometry, where a stands for the radius of the die hole; R stands for the radius of the punch head; t stands for the thickness of the specimen; k_sp_ is the correlation factor.

(6) $$\frac{F_{\mathrm{sp}}}{\sigma }=3.332k_{\mathrm{sp}}a^{-0.202}R^{1.192}t$$

The release of the CEN (European Committee for Standardization), small punch creep testing code of practice, facilitated the progress in the field of the small punch creep test. The above Equation (6) was recognized and widely used by many researchers. Blagoeva, Hurst [11] applied the CEN code to investigate the properties of welded P91 pipe. The creep behavior of the disc specimen was extracted from the weld P91, in which a desirable outcome was achieved. In this paper, for the most commonly used small punch test apparatus both in Europe and China, h = 0.5 mm, R = 1.25 mm and a = 2 mm. Thus, the expression leads to:

(7) $$\frac{F_{\mathrm{sp}}}{\sigma }=1.894k_{\mathrm{sp}}$$

Since the ratio between the applied load F_sp_ and equivalent stress σ is expressed in a linear relation, the slope k_sp_ lies in the key position in Equation (7). The research by Tettamandi and Crudeli [12] showed that the typical value for F_sp_/σ is in the range between 1.95 and 2.06. However, Bicego reported different result that F_sp_/σ ≈ 1.87 [13], In the research of Jeffs [14], the ksp value of 0.6 and 0.8 was determined for one single crystal material at the temperature of 950 °C and 1050 °C. In the research work of Li [15] and Feng [16], the value of k_sp_ = 1.0 (F_sp_/σ = 1.89) was adopted. However, the reasons of adopting this k_sp_ value were not well addressed. What’s more, the F_sp_/σ values adopted by the above researchers are empirical values. Not enough support from the experiment, which combines the uniaxial creep test and the small punch creep test, is found in their work.

Alegre [17] proposed a method to determine the value of k_sp_ based on an assumption that the ratio of the applied load F_sp_ and the equivalent stress σ follows a linear relation. The proposed method can be concluded as follows: for the uniaxial creep test at a specific testing temperature, when the applied stress σ approaches the creep strength of the material ($\sigma \to \sigma_{c}$), the failure time $t_{f}$ approached 0 ($t_{f}\to 0$) and the minimum strain rate ${\dot{\varepsilon }}_{\min}$ approaches ∞ (${\dot{\varepsilon }}_{\min}\to \infty$). For the small punch creep test, in an experiment of the same material under the same temperature, when the applied load F_sp_ approaches the maximum load (F_max_) of the test condition (F_sp_ → F_max_), at this critical moment, the small punch creep failure time $t_{f}$ approaches 0 ($t_{f}\to 0$) and the small punch minimum strain rate ${\dot{\varepsilon }}_{\min}$ approaches ∞ (${\dot{\varepsilon }}_{\min}\to \infty$). Under this condition the value of the correlation factor k_sp_ can be defined as:

(8) $$\frac{F_{\mathrm{sp}}}{\sigma }=\frac{F_{\max}}{\sigma_{c}}=1.89\;\times \;K_{\mathrm{sp}}$$

The above method by Alegre may sound reasonable; on the other hand, it is hard to define and measure the failure time when it approaches 0, and to what extent the minimum creep strain rate can be regarded as infinite. What’s more, this method uses the data collected at one single maximum load, which is not representative enough for different loading conditions.

In this paper, a new approach for determining the correlation factor was proposed. Both the small punch creep test and uniaxial creep test experiment was studied and compared. The k_sp_ value of different materials was derived, and suggestions for determining the stress of different materials are given, which provide a useful guidance for the research in the field of the small punch creep test.

## 2. Creep Model

### 2.1. Power Law Relations

In most of the life assessment system, it is the stress which is set as the control variable. Norton creep law [18] was first introduced in 1929, and was still the most commonly used one till now. Expressed in Equations (9) and (10), where $\dot{\varepsilon }$ stands for minimum creep strain rate, σ stands for the applied stress, $t_{R}$ stands for rupture time, A ($A^{\prime }$) and n ($n^{\prime }$) are power law parameters. power law relation, which consists of Norton creep law and rupture time vs. stress relation, gives the relationship between the stress versus the strain rate and stress versus rupture time. The minimum strain rate and the rupture time obtained by power law relations could be applied to investigate the residual creep life of the component. However, power law relations cannot be applied to characterize all creep properties; for engineering materials, it is strain rate and rupture time dependent properties that are of the greatest significance.

(9) $$\dot{\varepsilon }={A\sigma }^{n}$$

(10) $$t_{R}=A^{\prime }\;\times \;\sigma^{n^{\prime }}$$

Feng and Guan [16], Li [15] had also employed the power law relations to obtain creep properties by the small punch test. The stress and strain of the small punch test were derived, together with the stationary creep strain rate and rupture time, and the creep properties of the material can be derived.

### 2.2. Larson-Miller Parameters Method

Based on the Arrhenius equation, the Larson-Miller Parameters Method [19] gives the relation between stress, temperature and rupture time and it was widely used in life prediction. The Larson-Miller parameter is defined as follows:

(11) $$\mathrm{LMP}=T\left(C+\log t_{f}\right);\;C=20$$

where T stands for temperature, C is a material constant, $t_{f}$ stands for time to failure.

Ule [20], Hou [21] applied the Larson-Miller parameter method to interpret creep results of small punch test data, which shows that the Larson-Miller parameter method could be used to describe well the small punch creep results.

### 2.3. Monkman-Grant Method

The Monkman-Grant [22] model is a predictive model, which gives the relationship between the creep failure time $t_{f}$ and minimum strain rate ${\dot{\varepsilon }}_{\min}$. The Monkman-Grant relation is expressed as Equation (12), where C and m are material constant; it was initially developed for uniaxial creep experiments.

(12) $${\mathrm{logt}}_{f}+m\log\dot{\varepsilon }=C$$

Milicka [23], Dobes [24] and Hou [21] carried out studies to apply the Monkman-Grant equation to the small punch test. The researches indicate that the Monkman-Grant relation could be used to describe the creep behaviour of the small punch creep test.

## 3. Procedure of the Determination of the Correlation Factor

In this paper, a new approach for the determination of the correlation factor ksp was proposed. Since the stress state difference in the uniaxial creep test and the small punch creep test leads to the discrepancy in the creep results. The strain and the stress state of the small punch creep test needs to be converted into the form of a uniaxial creep test. The work done by Charkrabarty, Yang and Wang and “A Code of Practice for Small Punch Creep Testing” fulfilled the mentioned task. By adopting the similar Equation (3), the deflection of the lower face of the specimen δ, which can be easily obtained through an experiment, can be converted to the strain. Inspired from the “Europe Code of Practice: A Code of Practice for Small Punch Creep Testing”, the applied load of the small punch test F_sp_ can be converted into the stress by employing the Equation (6).

With the knowledge of the stress and strain of both the small punch creep test and the uniaxial test, comparison between the two tests can be made based on different creep models. Here, the procedure for determination of the correlation factor consists of the following three steps:

- Conduct both the small punch creep test and the uniaxial creep test under different stress levels
- Derive the strain and stress of the small punch test from the test curve
- Select creep models under different k_sp_ and compare the results of the small punch test and the uniaxial test and find out the best fit k_sp_ value

In accordance to the results of the experiment, the creep models include power law relations (Norton creep law, the rupture time vs. stress) and Larson-Miller parameter method.

## 4. Experiment Procedure

### 4.1. Materials and Preparation of Test Specimens

In this paper, three materials; P91, 1.25Cr0.5MoSi and 16Mo3, were used in the experiment. The above three materials are widely used in power plants, pressure vessel and piping which works at an elevated temperature. The chemical composition of P91 is (wt %) C 0.09, Cr 8.48, Mo 0.96, V 0.23, Nb 0.08, Mn 0.46. The chemical composition of 1.25Cr0.5MoSi material is (wt %) C 0.13, Mn 0.43, P 0.006, S 0.006, Si 0.068, Cr 1.29, Mo 0.49, Ni 0.13. The chemical composition of 16Mo3 material is (wt %) C 0.13, Mn 0.53, P 0.006, S 0.006, Si 0.28, Cr 1.06, Mo 0.29, Ni 0.23.

### 4.2. Small Punch Creep Test

**Figure 2:**
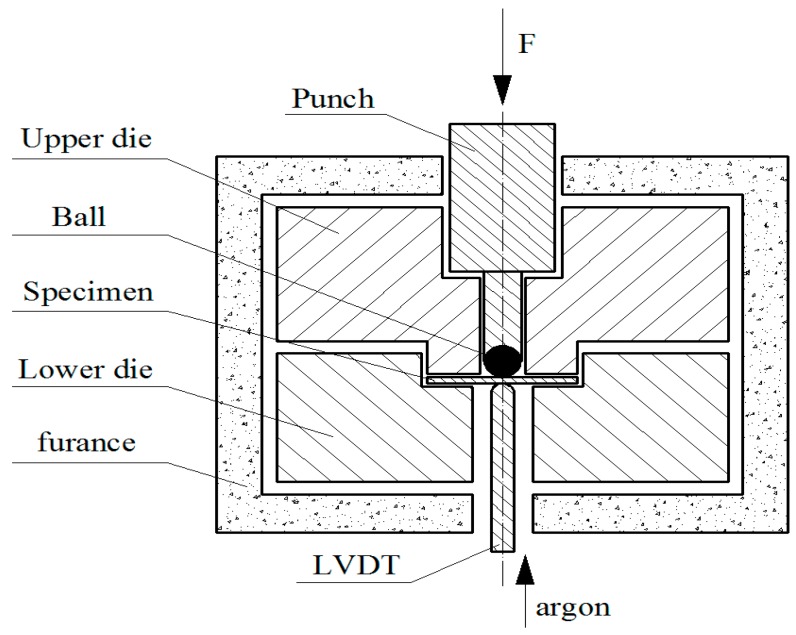
Principle sketch of the small punch test device.

The small punch test device is made up of four main parts; Figure 2 illustrates the principle sketch of small punch test device. The punch is a ceramic ball with a diameter of 1.25 mm; the upper and the lower die serve as the fixture of the specimen and restrict the motion of the specimen in these two directions; the displacement of the lower surface of the specimen was detected by the displacement sensor (LVDT) and was then recorded by a computer. The furnace heats the whole system inside and maintains the temperature at a stable value. The argon was used to prevent the oxidation of the specimen.

**Figure 3:**
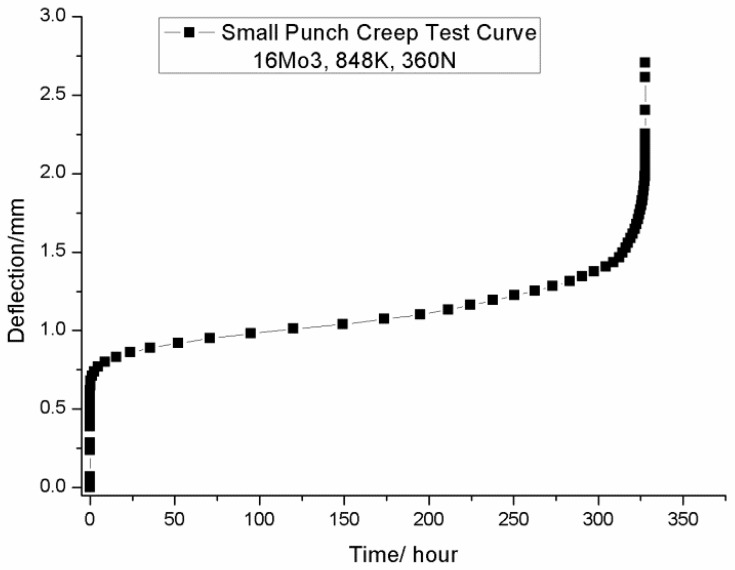
Typical small punch creep test curve.

The small punch tests of P91 and 1.25Cr0.5MoSi were conducted in East China University of Science and Technology in China and the small punch tests of 16Mo3 were conducted in Ostrava University of Technology in Czech Republic. The test temperature of P91 is 566 °C (839 K) and the applied load of the small punch creep tests were 430 N, 530 N and 630 N. The test temperature of 1.25Cr0.5MoSi is 550 °C (823 K) and the applied load of the small punch tests were from 370 N to 570 N. The test temperature of 16Mo3 is 575 °C (848 K) and the applied load of small punch creep tests were from 300 N to 480 N. The typical creep curve of the small punch creep tests was presented in Figure 3. Similar to the conventional uniaxial creep test, the small punch creep curve shows three distinct creep stages: the primary stage, the secondary stage and the tertiary stage.

### 4.3. Uniaxial Test

Uniaxial tests of the three above materials (P91, 1.25Cr0.5MoSi, 16Mo3) were carried out at constant temperatures (566 °C (839 K), 575 °C (848 K), 550 °C (823 K)), which are corresponded to the small punch test to make the comparison. The stress level of P91 was 135 MPa–180 MPa. The stress level for 1.25Cr0.5MoSi was from 100 MPa to 180 MPa and the stress level for 16Mo3 was from 160 MPa to 260 MPa. The stress levels for the creep experiment were all below the yield strength of the material.

## 5. Results and Discussion

The small punch creep test curves are time–deflection curves, which cannot be studied directly. Inspired from Equation (3), a similar procedure was conducted with the geometry of the applied small punch devices with a = 2 mm and R = 1.25 mm. Thus the relationship between deflection δ and strain ε in this case is expressed as:

(13) $$\varepsilon =0.23381\delta^{2}+0.40616\delta$$

**Figure 4:**
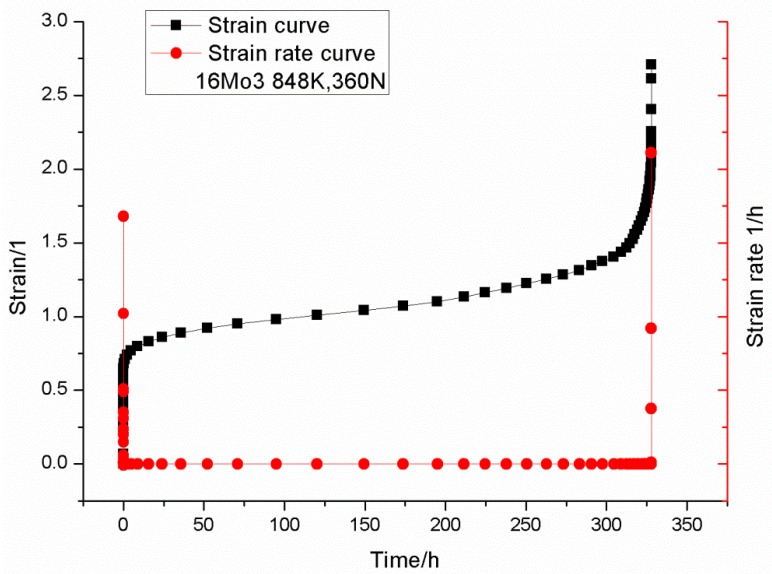
The time–strain versus strain rate curve of 16Mo3 at a testing condition of 848 K, 360 N.

By adopting the Equation (13), the time–deflection curve can be converted into a time–strain curve. Through differentiating the time–strain curve, the strain rate can be derived. Figure 4 shows the time–strain versus strain rate curve of 16Mo3 at a testing condition of 848 K, 360 N. The minimum creep strain rate ${\dot{\varepsilon }}_{\min}$ and the failure time $t_{f}$ of the small punch creep test can be obtained from the test curve. Similar to the conventional uniaxial creep test, a steady state creep strain rate can be observed in the secondary creep stage.

For P91, small punch tests and uniaxial tests were conducted under a different load; the minimum creep strain rates under each load or stress levels were listed in the Table 1.

**Figure 5:**
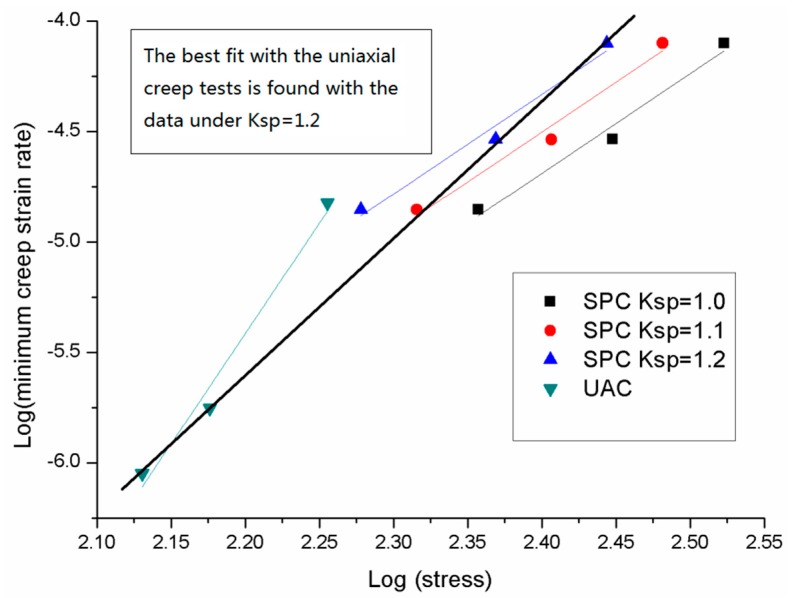
The Norton fitting results of both uniaxial creep results and small punch results of P91.

The Norton fitting results of both small punch creep tests under different k_sp_ values and uniaxial tests were plotted (log scale) in Figure 5. It could be derived from the figure that the minimum creep strain rate and stress of both small punch tests and uniaxial tests obey the Norton creep law. Different k_sp_ values of the small punch tests were compared; among all the three k_sp_ values, k_sp_ = 1.2 was suggested as the best fit. A good agreement was found between the uniaxial results and small punch creep test results under the k_sp_ value of 1.2.

**Figure 6:**
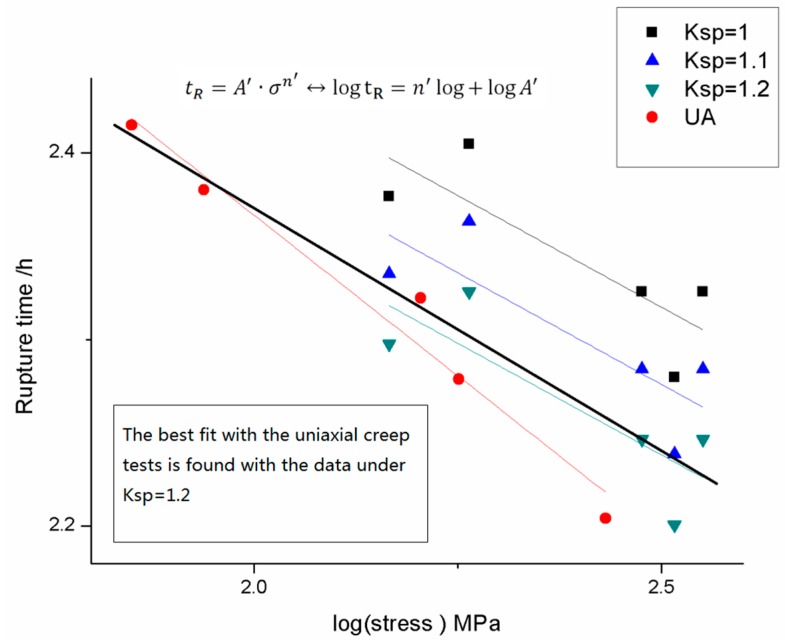
The rupture time versus stress fitting results of the small punch creep tests and the uniaxial tests of 16Mo3.

For 16Mo3, small punch tests were conducted under a load level from 300 N to 600 N, the fracture time of the small punch test and the uniaxial creep test under each loading condition was listed in Table 2 below. The fracture time versus stress fitting results of both small punch tests and uniaxial tests were plotted in Figure 6. It could be derived from the figure that the k_sp_ value of 1.2 predicts a good agreement in rupture time between small punch creep tests and uniaxial tests.

**Figure 7:**
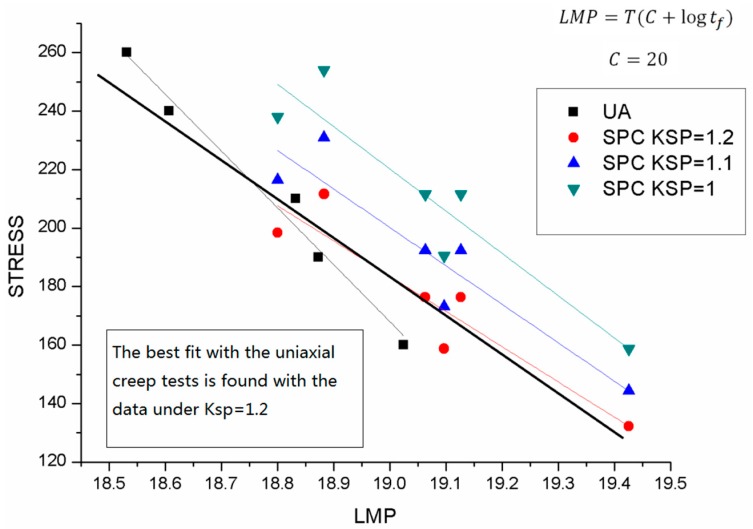
The Larson-Miller fitting results of both the small punch creep test and the uniaxial creep test of 16Mo3.

The Larson-Miller parameter method was also employed to investigate the results of 16Mo3, with the material constant C = 20; the Larson-Miller parameters of each load level of the small punch tests were derived. It can be seen from the Figure 7 that the k_sp_ value of 1.2 predicts a good agreement between uniaxial results and small punch creep results.

For 1.25Cr0.5MoSi, the small punch creep tests under different load levels and the uniaxial creep tests were conducted. The minimum creep strain rates under each load or stress levels were listed in Table 3.

**Figure 8:**
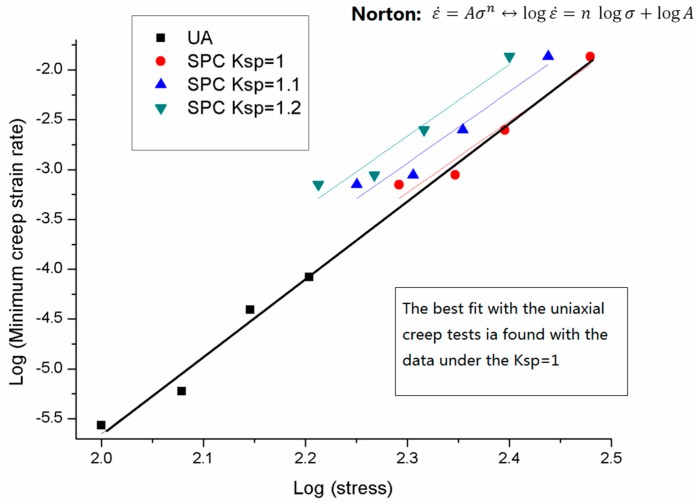
The Norton fitting results of the uniaxial creep results and the small punch results of 1.25Cr0.5MoSi.

The Norton fitting results of both small punch creep tests under different ksp values and uniaxial tests of 1.25Cr0.5MoSi were plotted (log scale) in Figure 8. It could be derived from the figure that the minimum creep strain rate and stress of both small punch tests and uniaxial tests obey the Norton creep law. Different k_sp_ values of small punch tests were compared; among all the three k_sp_ values, k_sp_ = 1 was suggested as the best fit. A good agreement was found between the uniaxial results and the small punch creep test results under the k_sp_ = 1.

From the above experiment, the fitting results of the power law relation and Larson-Miller indicates that: for the P91 and 16Mo3 experiment, k_sp_ = 1.2 is the best fit of uniaxial creep test and small punch creep test; for 1.25Cr0.5MoSi, k_sp_ = 1.0 is the best fit of uniaxial creep test and small punch creep test.

To sum up for the correlation factor study, k_sp_ values for different materials can be found by employing the stress related small punch creep model. The mentioned stress related creep model includes power law relations (Norton creep model, time versus stress model), and the Larson-Miller method. The obtained k_sp_ value for P91 and 16Mo3 is k_sp_ = 1.2 (F_sp_/σ = 2.268), and the k_sp_ value for 1.25Cr0.5MoSi is k_sp_ = 1.0 (F_sp_/σ = 1.89), which suggests that k_sp_ value varies with different materials’ conditions rather than a fixed value. Therefore, for a specific material, before applying the stress of the small punch creep test, the k_sp_ value needs to be obtained by an experiment.

What’s more, in Figure 6 and Figure 7, the fitting results showed some difference in low load and high load section, which may indicate the potential load dependence. Future research, which includes carrying out a small punch creep test at a distinct low load and high load level, may be needed to take a closer look into this issue.

## 6. Life Prediction

### 6.1. Monkman-Grant

The Monkman-Grant relationship for 16Mo3 was established to make the life prediction. When employing the Monkman-Grant relation, seven short time experiment sets were selected and the minimum creep strain rate and time to fracture data was listed in Table 4.

**Figure 9:**
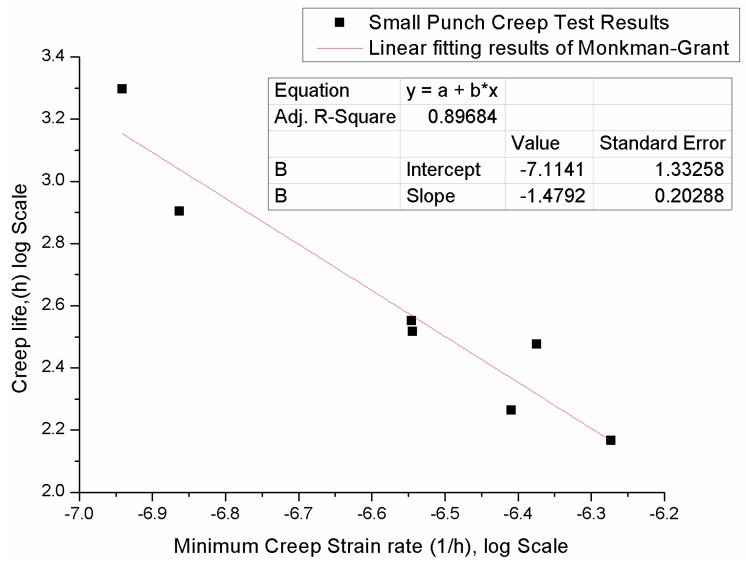
The small punch creep fitting results of the Monkman-Grant relation of 16Mo3.

The small punch creep data was plotted in a logarithmic scale; the fitting results of the Monkman-Grant relation are shown in Figure 9.

The linear fitting result is expressed as:

(14) y=−1.4792x−7.1141

where y stands for $\log t_{f}$, x stands for $\log\dot{\varepsilon_{\min}}$.

The error of the linear fitting can be defined as:

(15) $$\text{Error}=\frac{t_{p}-t_{f}}{t_{f}}\times 100\%$$

where $t_{p}$ stands for predicted life, and $t_{f}$ stands for experiment rupture life. The predicted life and the error of the prediction can be found in Table 5 below.

The average absolute error of the Fitting is 19.7%. The Monkman-Grant relation of the small punch creep test of 16Mo3 was established and a life prediction was made. Overall, the small punch creep test results of the material 16Mo3 fit the Monkman-Grant relation well, with the creep life decreasing with the increasing creep strain rate. The highest predicted error of 36.1% was seen in the loading condition of 330 N, and minimum error of 0.2% was seen in the loading condition of 450 N.

### 6.2. Larson-Miller Method

The Larson-Miller method was also employed to make the life prediction of 16Mo3; the Larson-Miller equation gives out a relation between the LMP and the applying load for a specific temperature.

(16) $$\mathrm{LMP}=T\left(C+\log t_{f}\right)\times 10^{-3}$$

**Figure 10:**
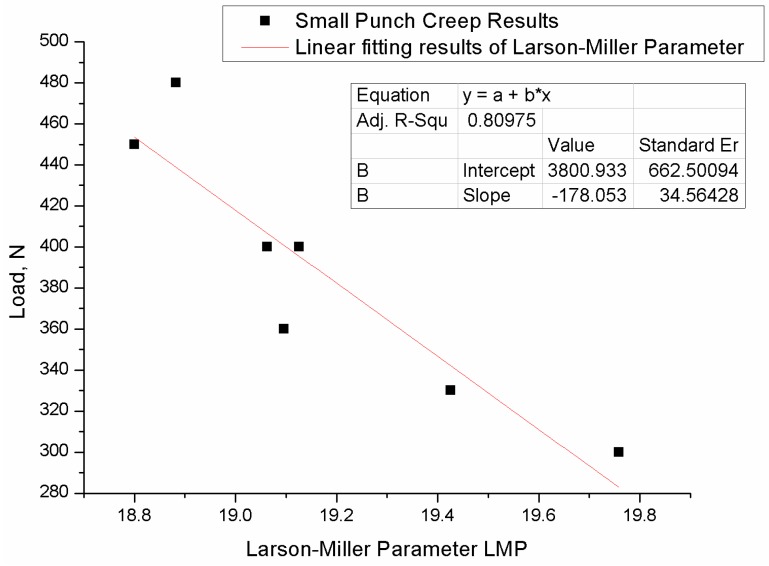
The Larson-Miller fitting result of the small punch creep test of 16Mo3.

The relationship between load and LMP was plotted in Figure 10. From the figure, it can be seen that the value of LMP decreases with the increasing load.

The fitting result is:

y = −178.053x + 3800.933
(17)

where x stands for LMP, y stands for applying load. The predicted life and the error of the prediction can be found in Table 6 below:

The predicted life under the loading condition of 480 N and 360 N showed a high error with the experiment life. However, the other data under the loading condition of 400 N and 450 N showed a high accuracy.

Typically, under the same test temperature, the rupture time shall decrease with the increase of the load. However, the data (360 N, 327.8 h), (480 N, 183.6 h) showed the inverse tendency. The experiment life under the load of 360 N is 327.8 h, which is shorter than the experiment life (355.5 h) under the load of 400 N. In addition, the experiment life under the load of 480 N is 183.6 h, which is longer than the experiment life (146.5 h) under the load of 450 N. The relative high error and the discrepancy in predicted life might be influenced by the following factors:

1. The friction between the specimen and the apparatus.
2. The non-uniform material properties which are easy to occur within the small scale specimen (8–10 mm × 10 mm × 0.5 mm).

The Larson-Miller relation of the small punch creep test was established, and life prediction was made under this predictive model. For most of the experiment sets, the life prediction showed a reasonable accuracy. Two set of the experiment showed a relatively higher error, the accuracy of the prediction may be influenced by factors which include friction factors and non-uniform distribution of the material properties.

To sum up for the life prediction, the Monkman-Grant method, as one of the rate dependent methods, predicts rupture life with the strain rate of each test. While the Larson-Miller parameter, which considers the impact of stress and testing temperature, predicts creep life with different applying loads to the small punch creep test. Although a discrepancy in experiment life and predicted rupture life showed up in some data points with both the Monkman-Grant and Larson-Miller creep model, others showed a reasonable accuracy, which shows that the small punch creep test could be used to characterize the creep behavior of materials. However, the accuracy of the life prediction fluctuates. For future research in the life prediction, duplicate experiment tests for each loading condition may be needed to produce a stable result.

## 7. Conclusions

In this paper, uniaxial creep tests and small punch creep tests were conducted. Creep models, which include power law relations and Larsson–Miller, were used to correlate both the results. The Monkman-Grant relation of the small punch creep test was established and Larson-Miller parameters, under different loading conditions, were derived. The main conclusions are summarized below:

1. The test curve of the small punch creep test is similar to that of the conventional uniaxial test. The small punch creep test curve showed three distinct creep stages. A same stationary creep strain rate was observed during the small punch creep test.
2. Power law relations and Larson-Miller of both the small punch creep test and the uniaxial creep test were established. The stress dependence of the minimum creep strain rate and rupture life of the small punch creep test and the uniaxial creep test showed the same tendency, which indicates that the small punch creep test could be used to characterize creep properties in the same way as the uniaxial creep test.
3. A new approach for determining the correlation factor was suggested. The k_sp_ values for three typical, high temperature materials was studied. The k_sp_ value for P91 and 16Mo3 was 1.2, and the k_sp_ value for 1.25Cr0.5MoSi was 1.0. Different k_sp_ values were found in different materials, which indicate that, for a specific material, instead of regarding the correlation factor as a constant value, the k_sp_ value needs to be obtained by experiment in advance, before applying the stress of the small punch creep test.
4. The life prediction model based on the Monkman-Grant relation and Larson-Miller of the small punch creep test was established; the predicted life showed a reasonable accuracy with exception in some load levels. It indicates that both Monkman-Grant and Larson-Miller could be used to describe the creep behavior of a material by a small punch creep test. In addition, the small punch creep test has the potential for life prediction.

## Figures and Tables

**Table 1:** Minimum creep strain rates under each load or stress levels of small punch creep test and uniaxial creep test.

Small Punch Creep Test (566 °C)		Uniaxial Creep Test (566 °C)	
Load (N)	Minimum Creep Strain Rate	Stress	Minmum Creep Strain Rate
430	1.40 × 10^−5^	135	8.96 × 10^−7^
530	2.91 × 10^−5^	150	1.77 × 10^−6^
630	7.95 × 10^−5^	180	1.50 × 10^−5^

**Table 2:** The fracture time of the small punch test and the uniaxial creep test under each loading condition.

16Mo3 Small Punch Creep Test (575 °C) 848 K		16Mo3 Uniaxial Creep Test (575 °C) 848 K	
Load (N)	Time to Fracture	Stress	Time to Fracture
300	1978.6	160	270.0
360	327.8	190	178.4
400	299.1	210	160.1
450	146.5	240	86.8
480	183.6	260	70.8

**Table 3:** The minimum creep strain rates under each load or stress levels of 1.25Cr0.5MoSi.

Small Punch Creep Test (550 °C)		Uniaxial Creep Test (550 °C)	
Load (N)	Minimum Creep Strain Rate	Stress	Creep Strain Rate
370	2.14 × 10^−4^	100	3.69 × 10^−6^
420	4.63 × 10^−4^	140	3.90 × 10^−5^
470	8.81 × 10^−4^	160	8.34 × 10^−5^
570	20.3 × 10^−4^	180	1.50 × 10^−4^

**Table 4:** The minimum strain rate and time to fracture data of 16Mo3.

Load (N)	Time to Fracture (T_f_/h)	Log (t_f_)	Minimum Creep Strain rate $\dot{\varepsilon }$ (1/h)	$\log\dot{\varepsilon }$
300	1978.6	3.296	1.15 × 10^−7^	−6.941
330	801.0	2.904	1.37 × 10^−7^	−6.863
360	327.8	2.516	2.85 × 10^−7^	−6.544
400	299.1	2.476	4.23 × 10^−7^	−6.374
400	355.5	2.551	2.85 × 10^−7^	−6.545
450	146.5	2.166	5.33 × 10^−7^	−6.273
480	183.6	2.264	3.90 × 10^−7^	−6.409

**Table 5:** The prediction results of 16Mo3 by the Monkman-Grant Method.

Load (N)	Predicted Life (h)	Experiment Life (h)	Error (%)	Absolute Error (%)
300	1422.5	1978.6	28.1%	28.1%
330	1090.6	801.0	36.1%	36.1%
360	368.0	327.8	−12.2%	12.2%
400	206.2	299.1	31.1%	31.1%
400	369.2	355.5	−3.9%	3.9%
450	146.2	146.5	0.2%	0.2%
480	232.3	183.6	−26.5%	26.5%

**Table 6:** The prediction results of 16Mo3 by the Larson-Miller Parameter Method.

Load (N)	Predicted Life (h)	Experiment Life (h)	Error (%)	Absolute Error (%)
300	1523.2	1978.6	23.0%	23.0%
330	964.0	801.0	−20.4%	20.4%
360	610.2	327.8	−86.1%	86.1%
400	331.6	299.1	−10.9%	10.9%
400	331.6	355.5	6.7%	6.7%
450	154.7	146.5	−5.6%	5.6%
480	97.9	183.6	46.7%	46.7%
